# Dispersal biophysics and adaptive significance of dimorphic diaspores in the annual *Aethionema arabicum* (Brassicaceae)

**DOI:** 10.1111/nph.15490

**Published:** 2018-10-25

**Authors:** Waheed Arshad, Katja Sperber, Tina Steinbrecher, Bethany Nichols, Vincent A. A. Jansen, Gerhard Leubner‐Metzger, Klaus Mummenhoff

**Affiliations:** ^1^ School of Biological Sciences Royal Holloway University of London Egham TW20 0EX UK; ^2^ Department of Biology, Botany University of Osnabrück Barbarastraße 11 D‐49076 Osnabrück Germany; ^3^ Laboratory of Growth Regulators Centre of the Region Haná for Biotechnological and Agricultural Research Institute of Experimental Botany Academy of Sciences of the Czech Republic Palacký University 78371 Olomouc Czech Republic

**Keywords:** abscisic acid (ABA), *Aethionema arabicum*, bet‐hedging, dimorphic diaspores, dispersal by wind and water, environmental adaptations, fruit biomechanics, pericarp‐imposed properties

## Abstract

- Heteromorphic diaspores (fruits and seeds) are an adaptive bet‐hedging strategy to cope with spatiotemporally variable environments, particularly fluctuations in favourable temperatures and unpredictable precipitation regimes in arid climates.
- We conducted comparative analyses of the biophysical and ecophysiological properties of the two distinct diaspores (mucilaginous seed (M^+^) vs indehiscent (IND) fruit) in the dimorphic annual *Aethionema arabicum* (Brassicaceae), linking fruit biomechanics, dispersal aerodynamics, pericarp‐imposed dormancy, diaspore abscisic acid (ABA) concentration, and phenotypic plasticity of dimorphic diaspore production to its natural habitat and climate.
- Two very contrasting dispersal mechanisms of the *A. arabicum* dimorphic diaspores were revealed. Dehiscence of large fruits leads to the release of M^+^ seed diaspores, which adhere to substrata via seed coat mucilage, thereby preventing dispersal (antitelechory). IND fruit diaspores (containing nonmucilaginous seeds) disperse by wind or water currents, promoting dispersal (telechory) over a longer range.
- The pericarp properties confer enhanced dispersal ability and degree of dormancy on the IND fruit morph to support telechory, while the M^+^ seed morph supports antitelechory. Combined with the phenotypic plasticity to produce more IND fruit diaspores in colder temperatures, this constitutes a bet‐hedging survival strategy to magnify the prevalence in response to selection pressures acting over hilly terrain.

## Introduction

Diaspores – fruits and seeds – with specific dispersal abilities and germinabilities evolved to support the angiosperm life cycle in adaptation to the prevailing environment (Linkies *et al*., 2010; Baskin *et al*., 2014; Willis *et al*., 2014). Climate change can trigger species range shifts and local extinctions, and is a global threat to plant diversity (Thuiller *et al*., 2005; Walck *et al*., 2011). Examples of this include the fact that global warming shifts the timing of alpine plant germination to unsuitable seasons (Mondoni *et al*., 2012), and also that several weeds will exert additional pressure for crop–weed competition and its management (Ramesh *et al*., 2017). For many plant species, the dispersed seed or fruit is the only phase in its life cycle when it can travel, with the potential to carry the whole plant, population, or indeed the entire species (Kesseler & Stuppy, 2012). Diaspore dispersal therefore has far‐reaching demographic, ecological and evolutionary consequences (Robledo‐Arnuncio *et al*., 2014; Willis *et al*., 2014). Knowledge of the ways in which plants disperse – and acquire the characteristics necessary for successful dispersal – has therefore been the subject of much theoretical and empirical research, dating back to observations by Linnaeus (van der Pijl, 1982) and Ridley's (1930) seminal compilation of early dispersal studies. The diversity of morphological and biomechanical shapes and structures inherent in plant seeds and fruits is the result of the pursuit of different strategies for successful dispersal and appropriate germination timing (Baskin *et al*., 2014; Larson‐Johnson, 2016; Sperber *et al*., 2017; Steinbrecher & Leubner‐Metzger, 2017). These early life‐history traits are especially important for annual plants, as they can only restart a new cycle via regeneration from the seed.

Most plant species commit themselves to monomorphism (monodiaspory) as their life‐history strategy, producing seeds and fruits of a single type that are optimally adapted to the respective habitat (Donohue *et al*., 2010; Walck *et al*., 2011; Baskin & Baskin, 2014). Interestingly, many plant species evolved a heteromorphism (heterodiaspory) strategy (Imbert, 2002; Baskin & Baskin, 2014; Baskin *et al*., 2014), a phenomenon described by Venable (1985) as ‘the production by single individuals of seeds (or sometimes single‐seeded fruits) of different form or behaviour’. Diaspore heteromorphism is confined to 18 of 413 angiosperm families, with distinct properties having been observed not only in size, shape, and/or colour, but also in other morphological, biomechanical, and germination characteristics, the degree of dormancy, dispersal ability, mucilage production upon imbibition, and ability to form a persistent seed bank (Sorensen, 1978; Mandák & Pyšek, 2001; Imbert, 2002; Lu *et al*., 2010; Dubois & Cheptou, 2012; Baskin & Baskin, 2014; Baskin *et al*., 2014). Heteromorphic diaspore traits may function as a bet‐hedging strategy to cope with the spatiotemporal variability of unpredictable habitats (Slatkin, 1974; Venable, 1985). Species with heteromorphic diaspores are most commonly annuals in dry Mediterranean and desert habitats, or in other frequently disturbed and stressful environments (Imbert, 2002). Almost all heteromorphic species of the cold deserts of northwest China are annuals (Baskin *et al*., 2014). Diaspores that differ in dispersal ability and germinability allow annual species to escape the harshness and unpredictability of their habitat in space (via dispersal) and time (delayed germination via dormancy).

In the Brassicaceae family, diaspore heteromorphism has evolved independently in a few genera (Imbert, 2002; Baskin *et al*., 2014; Willis *et al*., 2014; Mohammadin *et al*., 2017). Distinct types of diaspore heteromorphism evolved in the genus *Cakile* (Cordazzo, 2006; Avino *et al*., 2012), in the desert annual *Diptychocarpus strictus* (Lu *et al*., 2010, 2015), and in *Aethionema arabicum* (Mühlhausen *et al*., 2010; Lenser *et al*., 2016; Mohammadin *et al*., 2017). The genus *Aethionema*, the sister lineage of the core Brassicaceae, is thought to have originated and diversified in the ecologically, altitudinally, and geologically diverse Irano‐Turanian region (Franzke *et al*., 2011; Jiménez‐Moreno *et al*., 2015; Mohammadin *et al*., 2017). The dispersal of *Aethionema* spp. correlates with local events, such as the uplift of the Anatolian and Iranian plateaus, the formation of major mountain ranges, and probably a climatic change in seasonality towards summer aridity. Contemporary phylogenetic and biogeographic analyses identified Anatolia (Turkey) as one of the world's hotspots of biodiversity, which includes *c*. 550 Brassicaceae species (Şekercioğlu *et al*., 2011; Jiménez‐Moreno *et al*., 2015; Mohammadin *et al*., 2017). Semiarid steppe (Central Anatolian Plateau) and Mediterranean climates with dry summers (southwest Anatolia) dominate the region. The varied topography creates microclimates by elevation, including in the major mountain belts, e.g. the South Anatolian Taurus, which surround the Central Anatolian steppe. Diaspore heteromorphism evolved at least twice within the genus *Aethionema*, and was associated with a switch to an annual life form (Mohammadin *et al*., 2017).

*Aethionema arabicum* is a small, diploid, annual, herbaceous species whose genome sequence is published (Haudry *et al*., 2013). An advantage of *A. arabicum* as a model system for diaspore heteromorphism is that it exhibits true seed and fruit dimorphism with no intermediate morphs. Two distinct fruit types are produced on the same fruiting inflorescence (infructescence): dehiscent (DEH) fruits with four to six mucilaginous (M^+^) seeds, and indehiscent (IND) fruits each containing a single nonmucilaginous (M^−^) seed (Lenser *et al*., 2016, 2018). Upon maturity, dehiscence of the DEH fruit morph leads to the dispersal of M^+^ seeds, while the IND fruit morph is dispersed in its entirety by abscission. Comparative analyses of the anatomy and physiology of M^+^ and M^−^ seeds, and the DEH and IND fruits that contain them, have shown a multitude of differences. These findings suggest different roles and mechanisms of the dimorphic diaspores in the dispersal and germination strategy of *A. arabicum*. While principal agents of diaspore dispersal include transport by wind (anemochory), water (hydrochory), animals (zoochory), and the plant itself (autochory) (Fahn & Werker, 1972), little is known about morph‐specific dispersal properties and the adaptive benefits of bet‐hedging mechanisms associated with heteromorphic diaspore dispersal. Thus, the structural, functional, and physiological differences in *A. arabicum* diaspores, underpinned by mucilage production (M^+^) and fruit coat (pericarp) restraint (IND), remain unknown.

In this study, we used a comparative approach to investigate how distinct biomechanical, hormonal, and ecophysiological properties of the diaspores (M^+^ seed vs IND fruit) influence their dispersal and ability to persist in the high‐elevational scree‐slope environments of Anatolia. By integrating pericarp biomechanics, flight aerodynamics, and phenotypic plasticity, we investigated the suitability of *A. arabicum* diaspores for dispersal by hydrochory and anemochory as two contrasting mechanisms. This is consistent with the distinct ABA content and germinability of the dimorphic diaspores. Elucidating the bet‐hedging dispersal strategies in *A. arabicum*, and the plasticity of this dimorphism, allows us to better understand the adaptive significance of dispersal prevention and promotion mechanisms.

## Materials and Methods

### Seed collection and plant growth

Mature plants of *Aethionema arabicum* (L.) Andrz. ex DC. were grown from accession ES1020 (obtained from Eric Schranz, Wageningen University and Research Centre). Plants were grown in Levington F2 compost with added horticultural grade sand (F2 + S), under long‐day conditions (16 h 20°C : 8 h 18°C, light : dark) in a glasshouse.

### Diaspore biometrics and aerodynamic properties

Sixty replicates each of M^+^ seeds, M^−^ seeds extracted from IND fruits, and IND fruits were used to quantify height, width, and depth. A Leica DFC480 digital camera system, leica applications suite (v.4.5), and imageJ (v.1.5i) were used to measure distances. The mean mass of single diaspores was determined using eight replicates of 100 individuals. Diaspore shapes were approximated as triaxial ellipsoids (M^+^/M^−^ seeds, prolate spheroids; IND fruits, oblate spheroids), in order to calculate surface area. The time taken (h) for cumulative germination to reach 50% of its maximum (*T*_50_) was obtained from seed germination experiments with freshly harvested mature fruits and seeds placed in Petri dishes containing two layers of filter paper, 3 ml of dH_2_O, and 0.1% (v/v) Plant Preservative Mixture (Plant Cell Technology, Washington, DC, USA). Plates were incubated in an MLR‐350 Versatile Environmental Test Chamber (Sanyo‐Panasonic, Osaka, Japan) at 14°C and 100 μmol s^−1^ m^−2^ constant white light, as described in Lenser *et al*. (2016).

### Measurement of ABA concentration

Endogenous ABA concentration was determined using the Phytodetek^®^ ABA enzyme immunoassay kit (Agdia Inc., Elkhart, IN, USA) according to the manufacturer's instructions. Five replicates of diaspores (50 mg) were frozen in liquid nitrogen, and ground to a fine powder for 30 s using a Precellys^®^ 24 tissue homogenizer (Bertin Instruments, Montigny‐le‐Bretonneux, France). ABA was extracted with a chilled solution of 80% (v/v) methanol, 100 mg l^−1^ butylated hydroxytoluene, and 0.5 g l^−1^ citric acid monohydrate.

### Quantification of fruit valve dehiscence

Mature DEH and IND fruits from dry (17% relative humidity, RH), high‐humidity (65% RH) and water‐sprayed (100% RH) plants were clamped into the jaws of a single‐column tensile testing machine (Zwick Roell ZwickiLine Z0.5, Ulm, Germany) configured with a 200 N load cell. Separation speed was set at 1 mm min^−1^. Force–displacement data were obtained using 30 (17% RH and 65% RH) and 40 (100% RH) replicates from mature main branch infructescences. Maximum force (*F*_max_) and the slope of the linear elastic element were obtained.

### Fruit, fruit valve, and seed resistance to raindrop impact

A raindrop test method was applied to mature fruits on dry and wet infructescences. The opening (1.5 mm diameter) of a burette was placed 50 cm above a single fruit or seed. The number of single raindrops (of mean mass 53.55 ± 0.97 mg) directly impacting the fruit/seed were counted until fruit abscission (in DEH and IND fruits) or seed detachment (in M^+^ seeds) occurred. One hundred replicates of each diaspore were tested.

### Dispersal ability in still air

The time required for 100 diaspores to fall individually from a height of 108 cm in a plastic tube (15 cm diameter) was measured using high‐speed (120 frames s^−1^) videos (Nikon Coolpix P100, Tokyo, Japan). The fall rate (m s^−1^) was calculated over this height.

### Dispersal ability in flowing air

Distances travelled by dispersing diaspores were determined following Lu *et al*. (2010). One hundred diaspores were individually exposed for 60 s to a constant stream of air parallel to a flat landing surface. A ventilator (Quigg BF‐12A, Hamburg, Germany) was used to simulate a wind velocity of 4 m s^−1^ in a custom‐built wind channel (height 60 cm, length 360 cm, width 48 cm). Diaspores were released from a height of 30 cm, 10 cm from the front of the ventilator, and the total distance travelled was measured to the nearest 0.01 m.

### Effect of diaspore on substrate attachment

Adherence potential was tested by placing diaspores on dry and water‐saturated sand (grains < 2 mm) in Petri dishes for 10 min. Seeds/fruits were rotated to allow whole‐surface contact with the substrate, and subsequently removed. The mass of the dry seeds/fruits, including the soil particles attached to them, was compared with the mass of the same dry seeds or fruits before exposure. Three replicates each of 25 diaspores were tested.

### Diaspore displacement mediated by surface water runoff

Diaspore displacement by surface water runoff was quantified using a custom‐built device consisting of a container with 53 holes (400 μm diameter) above a 6°‐sloped 80 × 30 cm plate, covered with 80‐grit sandpaper (modified from García‐Fayos *et al*., 2010). During each simulation, water flow was stopped after 1 l had been discharged from the sprinkling head within 2.5 min. The total distance travelled by 100 replicates of each diaspore on the plate was measured.

### Diaspore buoyancy

Three replicates, each of 25 diaspores, were placed on the surface of 150 ml of water contained in 250 ml Erlenmeyer flasks. Water movement in the flasks was simulated by agitation on an orbital shaker at 100 rpm (Truscott *et al*., 2006; Sun *et al*., 2012). The number of floating diaspores was counted over time.

### Statistical analyses

R was used to assess the distribution of the data and test for normality and homogeneity of variance. Normal and homogeneous data were subjected to one‐way ANOVA, with *post hoc* comparisons made by a Tukey's honest significant difference test. The rejection threshold for all analyses was *P *<* *0.05. Data exhibiting a nonGaussian distribution or nonhomogenous variances were transformed by a Box–Cox (Box & Cox, 1964) transformation using the mass package (Venables & Ripley, 2002) in R (nontransformed data are shown in all figures and tables). Analyses of diaspore behaviour on substrata were performed with a three‐way ANOVA (with diaspore type, imbibition state, and sand state as main factors). Analyses of diaspore buoyancy were performed with a univariate type‐III repeated‐measures ANOVA (with diaspore type and time interaction included as fixed effects). All statistical analyses were conducted in R (v.3.4.2; R Foundation for Statistical Computing, Vienna, Austria) or Graphpad prism (v.7.0a; San Diego, CA, USA).

## Results

### *Aethionema arabicum* habitat and dimorphic diaspore properties

**Figure 1:**
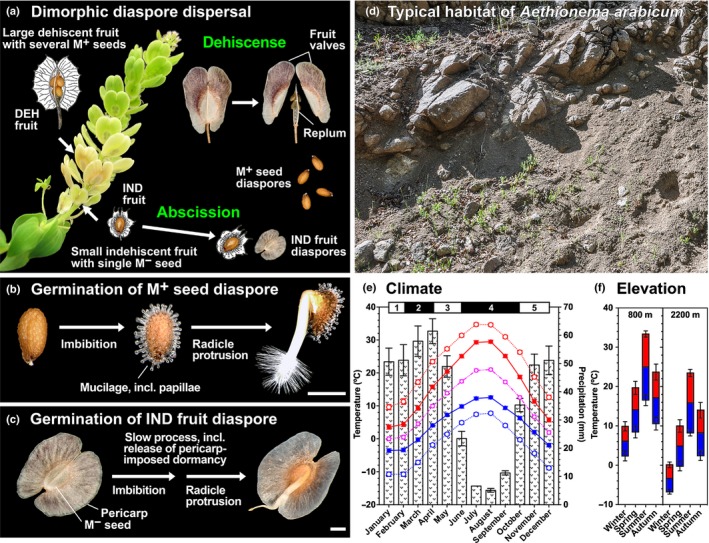
Dispersal, germination, habitat and climate of the dimorphic fruits and seeds of *Aethionema arabicum*. (a) *Aethionema arabicum* infructescence showing dehiscent (DEH) and indehiscent (IND) fruit morphs. Mature DEH fruits do not abscise from the mature infructescence, but instead disperse multiple mucilaginous (M^+^) seed diaspores. Mature IND fruit diaspores, containing a single, nonmucilaginous (M^−^) seed, disperse in their entirety from the mature infructescence. (b) Upon imbibition, the M^+^ seed diaspore produces mucilage from the outer cell walls of the seed coat epidermal layers, forming conical papillae of up to 200 µm. (c) By contrast, germination of the IND fruit diaspore is a slower process, in part mediated by pericarp‐imposed dormancy. A single M^−^ seed remains enclosed within the winged IND fruit, unless they are released by external mechanical means, and the radicle protrudes from within the pericarp. Bars, 1 mm. (d) Characteristic stony‐slope and steppe habitat of *A. arabicum*, taken in the Kırşehir Province in the central Anatolian region of Turkey. (e) Climate diagram of sites in Turkey from which *A. arabicum* has been collected. The maximum (solid red) and minimum (solid blue) temperatures of an average day for every month are shown. The averages of the hottest day (dashed red) and coldest night (dashed blue) of each month from the last 30 yr are also shown. The dashed magenta line indicates the mean monthly temperature. Mean monthly precipitation (bars) is shown. All climate data were obtained from Meteoblue (developed at the University of Basel, Switzerland, based on weather forecast models of the National Oceanic and Atmospheric Administration and National Centers for Environmental Prediction) from a total of 25 specimens archived at the Global Biodiversity Information Facility (GBIF.org, https://doi.org/10.15468/dl.w63b7k; accessed 31 January 2018). Data are means ± 1 SE. Numbers above the climate diagram indicate typical life cycle stages of diaspore persistence in seed bank (1,5), germination and seedling establishment (2), vegetative, flowering, and reproductive growth (3), and diaspore dispersal and plant senescence (4). (f) Seasonal variation of mean maximum (red) and mean minimum (blue) temperatures at low (800 m) and high (2200 m) elevations that support *A. arabicum* growth. Data are means ± 1 SE.

The dimorphism of *A. arabicum* is characterized by the M^+^ seed diaspores, dispersed by dehiscence, and by the IND fruit diaspores, dispersed by abscission (Fig. 1a). Various morphological properties that may be influencing M^+^ seed and IND fruit dispersal were all significantly different (Table 1). While freshly harvested mature M^+^ seeds germinated readily within 2 d at 14°C, IND fruit germination required at least 2–4 wk (Fig. 1b,c; Table 1). Further to this, most of the M^+^ seeds but none of the IND fruits germinated at 20°C, which suggests differences in dormancy and temperature responses of the dimorphic diaspores. This difference in diaspore germinability is mediated, at least in part, by a 34‐fold higher ABA concentration in the IND fruits than in the M^+^ seeds (Table 1). This difference is associated with an ABA concentration in M^−^ seeds that is double that in M^+^ seeds, combined with a very high ABA concentration of the pericarp. The low germinability of the IND fruits, therefore, seems to constitute a case of ABA‐mediated and pericarp‐enhanced dormancy. These differences, combined with the observed plasticity in DEH/IND fruit ratios and numbers (Lenser *et al*., 2016) and the unstudied dispersal mechanisms of its dimorphic diaspores, are expected to support the adaptation of *A. arabicum* in its natural habitat and climate.

**Table 1:** Comparative dispersal and biophysical properties of *Aethionema arabicum* dimorphic diaspores

	Diaspore		
	M^+^	IND	M^−^ a
Mass (mg)	0.34 ± 0.7^b^	1053.5 ± 2.4^a^	0.27 ± 0.6^c^
Surface area (mm^2^)	1.7 ± 0.2^c^	53.9 ± 9.9^a^	2.1 ± 0.3^b^
Height (μm)	1099 ± 66^b^	5764 ± 530^a^	1125 ± 67^b^
Width (μm)	580 ± 45^b^	4614 ± 527^a^	680 ± 55^b^
Depth (μm)	500 ± 56^a^	516 ± 51^a^	354 ± 26^b^
*T*_50_ at 14°C (h)	41.4 ± 0.8^b^	364.2 ± 10.2^a^ b	49.6 ± 7.4^a^
ABA (pmol per diaspore)	0.16 ± 0.06	5.47 ± 0.29^c^	0.34 ± 0.08

Dry mass, surface area, biometrics and germinability, and abscisic acid (ABA) concentration are shown for the multiple mucilaginous (M^+^) seed and indehiscent (IND) fruit diaspores. Note that nonmucilaginous (M^−^) seeds, obtained by mechanical removal from the IND fruits, were used as a comparison to reveal the roles of the IND pericarp.

a Not a diaspore in nature, extracted from IND fruit for experimental comparison.

b *T*_50_ for half of the population, while the other half remained dormant for at least 1 month (Lenser *et al*., 2016). *T*_50_, time taken for cumulative germination to reach 50% of its maximum. *n *=* *8, each with 100 replicate seeds (mass); 60 seeds (surface area, height, width and depth); three Petri dishes, each with 20 replicate seeds (*T*_50_); five each with 50 mg diaspores (ABA). ANOVAs were performed on each of the variables. The *F*‐statistic was significant for each ANOVA (*P *<* *0.05). Within each series for a given variable, values followed by the same letter do not differ (Tukey's honest significant difference, *P *<* *0.05).

c ABA concentration of one IND pericarp is 5.31 ± 0.25 pmol per diaspore.

*Aethionema arabicum* is described as a poorly competitive species, and typically grows in dry locations near fields, in steppes, and on stony slopes and screes with highly eroded calcareous substrate (Fig. 1d) (Davis, 1965; Babaç, 2004; Sunar *et al*., 2016; Delcheva & Bancheva, 2017; Mohammadin *et al*., 2018). In the Anatolian peninsula, it grows at 600–2700 m elevation. Using the locations of 25 accessions, we have generated climate diagrams for the seasonal changes in precipitation, minimal and maximal temperatures at average elevation, as well as at low and high elevations (Fig. 1e,f). Germination, seedling establishment, and vegetative growth occur early in spring, flowering in April to June, and fruit maturation and diaspore dispersal during the dry summer and the wetter autumn (Fig. 1e). The distinct morphology of the dimorphic diaspores (Table 1) suggests that they have distinct roles and biophysical dispersal mechanisms linked to the climatic regime of the region.

### Dimorphic fruit biomechanics

**Figure 2:**
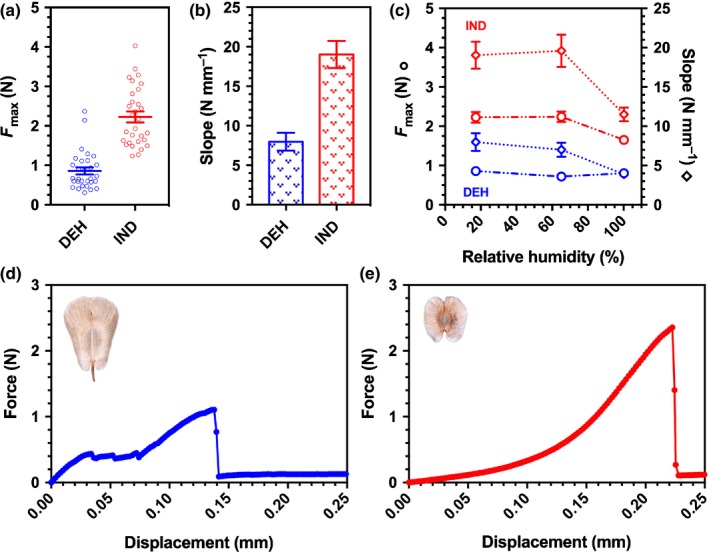
Biomechanics of dehiscent (DEH) and indehiscent (IND) *Aethionema arabicum* fruits. (a, b) Comparative maximum force (*F*_max_) (a) and slope (approx. modulus of elasticity) (b) required to separate fruit valves from fruits under dry conditions. (c) Comparisons of *F*_max_ and slope in 17%, 65% and 100% relative humidities show a trend towards gradual decrease in stiffness in both fruits, while *F*_max_ also decreases in the IND fruit but remains unchanged in the DEH fruit. (d, e) Characteristic force–displacement curves of mechanical tests in which fruit dehiscence occurred in *A. arabicum*, revealing distinct fracture biomechanical properties of slow, gradual failure of the DEH fruit (d), and sudden, complete failure for the IND fruit (e). *n *=* *30. Error bars ± 1 SEM.

A comparative biomechanical analysis of DEH and IND fruit valve separation at low and high RH values (simulating the dry summer and wetter autumn) revealed, first, that DEH and IND fruits differed fundamentally in their dehiscence behaviour (Fig. 2). Plant biomechanics is an integral component of the abiotic interactions of plants with gravity, wind and soil. Tensile tests determine the force needed to elongate a sample to its breaking point and provide insight into its material properties. The maximal force and the elasticity associated with separating the fruit valves were more than twofold lower for DEH than for IND fruits (Fig. 2a,b). DEH fruit force–displacement curves show a progressive failure with a sequence of force drops. This ‘composite’‐type failure for DEH fruits, together with the lower maximal force, demonstrates that dehiscence is indeed the M^+^ seed dispersal mechanism operating for this fruit morph. Dehiscence required to disperse the M^+^ seeds was triggered in any humidity condition (Fig. 2c). By contrast, the IND fruit diaspore, for which detachment by abscission is the dispersal mechanism (Fig. 1a), shows a completely different profile. After a preloading phase, the force–displacement curve shows a linear region and no distinct plastic deformation (‘brittle’‐type failure) (Fig. 2e). The maximal force and elasticity are dependent on the humidity conditions (Fig. 2c). The finding that they are significantly lower in high‐humidity conditions is consistent with the role of fruit valve splitting to aid radicle emergence during IND fruit germination (Fig. 1c). These contrasting biomechanical properties of the dimorphic fruits, therefore, support dehiscence and abscission, respectively, as distinct mechanisms for the M^+^ seed and IND fruit dispersal.

Also in agreement with IND fruit dispersal by abscission is the fact that the force required to remove dry IND fruits from *A. arabicum* plants is approx. six‐fold lower than that required to remove dry DEH fruits (Lenser *et al*., 2016). To obtain evidence that this is also the case in humid conditions, to aid dispersal in autumn when precipitation increases (Fig. 1f), we investigated the effects of direct water droplet impacts on fruit abscission. While only 6 (± 1) water droplets (mean ± SE) were required to detach a ‘wet’ IND fruit by abscission, for a ‘wet’ DEH fruit, 97 (± 9) water droplets were required. In rare cases where M^+^ seeds were still attached to the replum after DEH fruit valve detachment had occurred (Fig. 1a), this process was also aided by rain: only 9 (± 4) water droplets were required to detach an M^+^ seed from the replum and, in 70% of the cases, this was achieved by a single water droplet. Dispersal by rain (ombrohydrochory) is therefore a likely mechanism for the dispersal of both dimorphic diaspores, but seems more important for the abscission of the IND diaspore.

### IND fruits exhibit the greatest ability for wind dispersal

**Figure 3:**
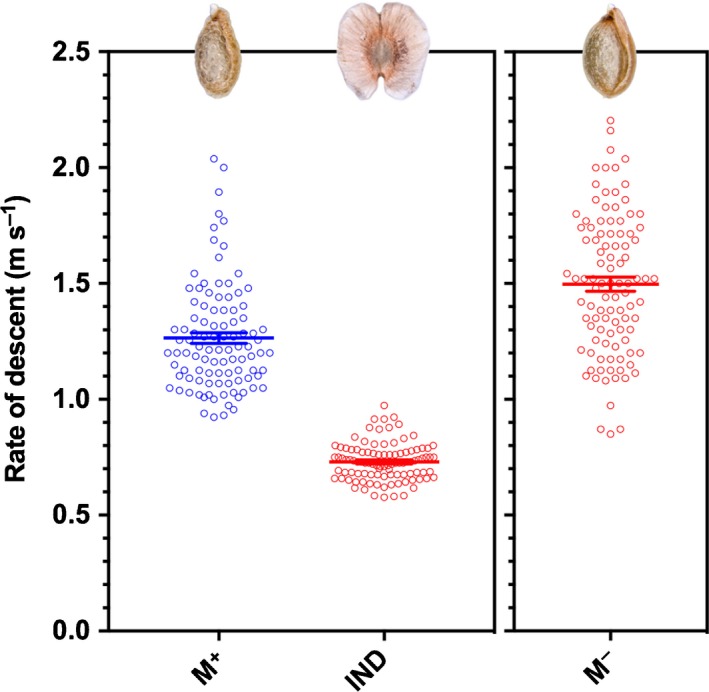
Comparative fall rates of *Aethionema arabicum* diaspores during descent. Mean rate of descent (m s^−1^) of multiple mucilaginous (M^+^) seed diaspores and intact indehiscent (IND) fruit diaspores when released from a height of 1.08 m in still air. IND fruits required greater time to fall, confirming that the pericarp can be regarded as an adaptation for wind dispersal. The fall rates of nonmucilaginous (M^−^) seeds (mechanically removed from IND fruits) are shown for comparison. Differences between M^+^ seed and IND fruit diaspores are significant (*P *<* *0.001). *n *=* *100. Error bars ± 1 SEM.

The wings, flat structure and large surface area (Fig. 1a,c; Table 1) of the IND fruit morph is indicative of an adaptation for dispersal by wind (anemochory). Mean descent rates (Fig. 3) of M^+^ seed and IND fruit diaspores, as well as for M^−^ seeds (used as comparison to reveal the roles of the IND‐pericarp), were significantly different from one another (*F*_2,297_ = 438.1, *P *<* *0.001). Intact IND fruits descended at the slowest rate, followed by M^+^ seeds (Fig. 3).

**Figure 4:**
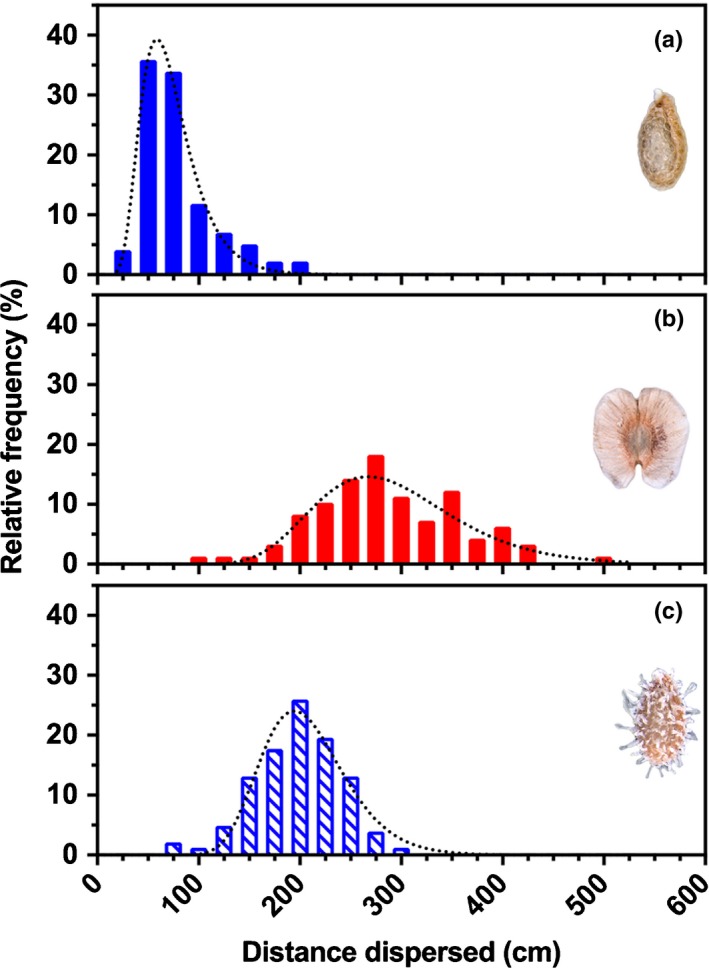
Frequency distribution patterns (‘seed dispersal shadows’) of the dispersibility of *Aethionema arabicum* diaspores from the mother plant. (a, b) The dispersibilities of dry multiple mucilaginous (M^+^) seed (a) and dry indehiscent (IND) fruit diaspores (b) differ significantly (*F*_2,313_ = 437.2, *P *<* *0.001) in a 4 m s^−1^ continuous current of air. M^+^ seeds achieve only short‐distance dispersal from the mother plant, while the longer‐distance dispersibility of IND fruit diaspores may influence colonization patterns in new steppe habitats. (c) Comparisons are made to rare cases where M^+^ seeds imbibe while still attached to the replum, and are subsequently redried and then dispersed. *n *=* *100. Dotted lines indicate log‐normal curves fitted to individual frequency distributions.

Quantified wind velocities in Anatolia are 1–4 m s^−1^ (Apaydin *et al*., 2011), and 4 m s^−1^ currents are typically used in such wind dispersal experiments. We found that, as expected, because of the wings, IND fruits (mean ± SD = 286.6 ± 7.2 cm) dispersed further than M^+^ seeds (78.4 ± 3.7 cm). In cases where M^+^ seeds imbibe while attached to the replum of DEH fruits (after fruit valve detachment) but do not disperse, redried M^+^ seeds were also included in the analysis. Dispersal of redried M^+^ seeds exhibited a ‘seed shadow’ with a mean distance of 197.3 cm (Fig. 4). These results demonstrate that IND fruits, with *c*. 3 m mean and *c*. 5 m maximum dispersal distance, exhibit the greatest ability for anemochorous dispersal. Although wind dispersal is more efficient over flat and uniform terrain, the IND fruit morph may have the potential for lateral and upward dispersal by gusts of wind in slope and scree habitats (Fig. 1d).

### Evidence for restricted secondary dispersal (antitelechory) in M^+^ seeds

**Figure 5:**
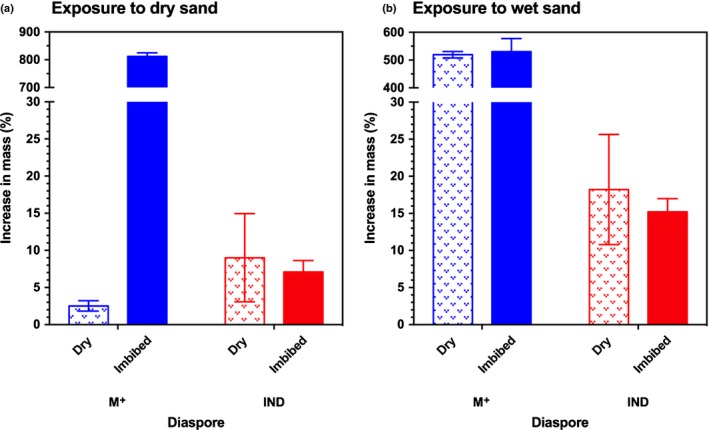
Comparative behaviour of *Aethionema arabicum* diaspores on fine‐grained sandy soil substrata. (a, b) Relative changes in mass of dry and imbibed multiple mucilaginous (M^+^) seed and indehiscent (IND) fruit diaspores upon exposure to dry (a) and water‐saturated (b) sand. By comparing the initial and final weights of the diaspores, a percentage was obtained by which the mass of the dispersal unit had been increased by adherent sand particles, thus illustrating the effectiveness of mucilage production in M^+^ seed diaspores as a means of antitelechory. The unchanged mass of IND fruits suggests the fruit diaspores retain high dispersibility. *n *=* *3, each with 25 replicates. Error bars ± 1 SEM.

Once dispersed from the mother plant (primary dispersal), the behaviour and interaction of diaspores with their substrata may restrict (antitelechory) secondary dispersal, a multistep process that further extends the dispersal distance from the parent plant. Mechanisms that thereby support antitelechory may explain the contrasting differences between M^+^ seed and IND fruit dispersal. Assessing the behaviour of dry and imbibed *A. arabicum* diaspores on sandy substrate, we found striking differences between the dimorphic diaspores regarding the adherence potential of sand particles and its effects on diaspore mass. Comparisons of the initial (without contact with sand) and final (with sand particles attached) masses of the diaspores showed there was a significant interaction between the effects of diaspore morph (M^+^ seed vs IND fruit), state (dry vs imbibed), and sand substrate (dry vs water‐saturated) on the relative increase in mass (*F*_2,24_ = 10.325, *P *<* *0.001). For M^+^ seeds, a striking increase in mass (*P *<* *0.001) was evident, while for IND fruits no such abundant adherence of sand particles was evident (Fig. 5). The comparison with M^−^ seeds demonstrated that this difference is a result of the adherence of substrate particles via the production of M^+^ seed coat mucilage (Figs 1b, 5). In contrast to M^+^ seeds, M^−^ seeds only produce a thin mucilage layer and the outer surface of the IND pericarp is mucilage‐free (Fig. 1c). The striking increase in M^+^ seed mass therefore restricts its secondary dispersal and constitutes a mechanism for antitelechory.

**Figure 6:**
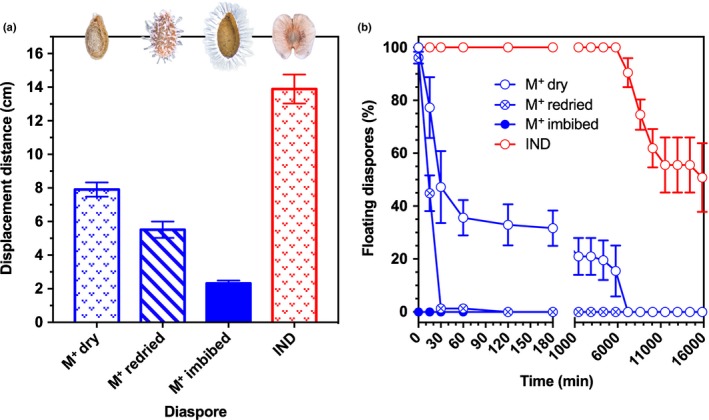
Surface water runoff displacement and buoyancy of *Aethionema arabicum* fruit and seed diaspores. (a) Distance displaced by surface water runoff across a sloped sandpaper plate of dry, redried and imbibed multiple mucilaginous (M^+^) seeds, in comparison to indehiscent (IND) fruits. Nonmucilaginous (M^−^) seeds (not shown), when manually excised from the IND fruit, were displaced by 32.3 ± 1.9 cm. *n *=* *100. Error bars ± 1 SEM. *Post hoc* pairwise comparisons confirmed that the dimorphic diaspores were statistically different from each other (*P *<* *0.001). (b) Buoyancy of *A. arabicum* seed and fruit diaspores. Dry M^+^ seeds, redried M^+^ seeds, and imbibed M^+^ seeds show progressive sinking as a result of mucilage extrusion. IND fruit diaspores, by contrast, start to sink after 5 d of shaking, while all M^−^ seeds (not shown) remained floating at the end of the experimental treatment. Symbols are offset on the *x*‐axis for clarity. *n *=* *3, each with 25 replicates. Error bars ± 1 SEM.

To investigate the mechanisms via which *A. arabicum* diaspores may promote or restrict dispersal by water currents (nautohydrochory), we simulated diaspore displacement by surface water runoff events using a sandpaper‐sloped surface. The distances travelled varied significantly among diaspores (*F*_4,495_ = 143.3, *P *<* *0.001) (Fig. 6a). The IND fruit morph was 1.8‐ to six‐fold further displaced compared to the M^+^ seed morph in its different states (dry, redried, imbibed). The comparison with the nonmucilaginous M^−^ seed, which is similar in size and shape (Table 1), demonstrates that the reduced nautohydrochoric properties of the M^+^ seed diaspore are also a result of seed coat mucilage extrusion.

As IND fruit dispersal does not appear to be restricted by surface water runoff, we wanted to compare the buoyancy potential of the *A. arabicum* dimorphic diaspores. There was a highly significant time × diaspore type interaction (*F*_64,160_ = 24.84, *P *<* *0.001; Fig. 6b) over the experimental period. The mean percentage of floating diaspores across 11 d differed significantly between M^+^ seeds in different states (dry, redried, imbibed) and IND fruits (*F*_1,364_ = 4.3, *P *<* *0.05). Most marked differences between diaspores occurred within the first 30 min; all redried and imbibed M^+^ seeds were sinking rapidly, and only *c*. 45% of the dry M^+^ seeds (but 100% of the IND fruits) remained floating (Fig. 6b). Dry M^+^ seeds were progressively sinking (0% floating after *c*. 4 d), while IND fruits remained floating for many days (100% after 4 d, and > 50% after 11 d; Fig. 6b). Taken together, this comparison strongly suggests that IND fruits are adapted for dispersal in space (telechory) and time (pericarp‐mediated dormancy), whereas M^+^ seeds possess mechanisms to remain in the direct vicinity of mother plants (antitelechory).

## Discussion

### Distinct dispersal and dormancy mechanisms of dimorphic diaspores

Our biomechanical, ecophysiological, and morphological comparison of the *A. arabicum* dimorphic diaspores revealed that they correspond to distinct abiotic dispersal modes and agents. The biophysical properties of the M^+^ seed diaspore support antitelechoric mechanisms to anchor the dispersed M^+^ seed in the direct vicinity of the mother plant. By contrast, the biophysical properties of the winged IND fruit diaspore support telechoric mechanisms (by wind and water), favouring local population dispersal over longer distances. Whereas diaspore dispersal of monomorphic species can only employ the dispersal mode evolved for their single diaspore type, heteromorphic species have evolved an array of distinct dispersal and dormancy adaptations, proposed to provide a bet‐hedging strategy to cope with the spatiotemporal variability of their unpredictable habitats (Imbert, 2002; Baskin *et al*., 2013, 2014; Willis *et al*., 2014; Lu *et al*., 2015). In the Brassicaceae *Cakile* spp. and *Diptychocarpus strictus*, each fruit is fragmented to give rise to different morphs and, therefore, the ratio between the morphs is developmentally constrained (Cordazzo, 2006; Lu *et al*., 2010, 2015). By contrast, the *A. arabicum* dimorphic diaspores derive from distinct fruits, and both the diaspore ratios and numbers can change in response to ambient temperature during reproduction (Lenser *et al*., 2016). The *A. arabicum* dimorphic system hence provides a blend of bet‐hedging and plasticity, which allows it to modulate dispersal ability and germinability in response to environmental cues. We discuss here how this relates to the native habitat and climate (Fig. 1), and reveal properties of its diaspores as adaptations to distinct dispersal mechanisms.

That ABA is a key hormone, mediating the distinct environmental responses to control germination timing by dormancy mechanisms, is well established (Finch‐Savage & Leubner‐Metzger, 2006), but nothing was known about differences in the ABA content of dimorphic diaspores in *A. arabicum*. We found that a 34‐fold higher endogenous ABA concentration in the IND fruit diaspore compared with the M^+^ seed diaspore is consistent with the low germinability (high degree of dormancy, HDo) of the IND fruit and the higher germinability (low degree of dormancy, LDo) of the M^+^ seed diaspore. Also, the ABA concentrations in M^−^ seeds were higher than in M^+^ seeds, but much of the ABA was contained in the pericarp. High ABA concentrations controlling germination timing are also known from dry fruits of monomorphic species (Benech‐Arnold *et al*., 1999; Hermann *et al*., 2007; Chen *et al*., 2008). The finding of high ABA concentrations in the IND pericarp is consistent with a key role in the pericarp‐mediated dormancy mechanism.

### M^+^ seed diaspore properties support antitelechory

Flowering and reproductive development in April–June lead to diaspore dispersal during the dry summer and wetter autumn (Fig. 1e), after which the (annual) mother plant dies. In agreement with the idea that these conditions aid dispersal, our biomechanical analysis shows that M^+^ seed dispersal via dehiscence (fruit valve separation leading to fruit opening) of DEH fruits occurs easily in both dry and humid conditions (Fig. 2). Fruit opening by dehiscence is the default trait in the Brassicaceae (Mühlhausen *et al*., 2013), and dehiscence upon wetting has generally been associated with plants adapted to arid environments (Gutterman, 2002; Pufal *et al*., 2010). Our finding that *A. arabicum* DEH fruit dehiscence can occur in both dry and humid conditions supports the view that M^+^ seed dispersal can be temporally staggered from the late dry summer to the wetter autumn. Upon DEH fruit dehiscence, the majority of M^+^ seeds detach from the replum and undergo primary dispersal in close vicinity to the mother plant. Together with their lower ABA concentration and dormancy, germination of the M^+^ seeds will ensure the progeny sustains its presence in favourable locations.

The phenomenon of mucilage production, known as myxospermy, is commonly understood to serve as an anchorage and adherence mechanism for seed retention; it is of particular importance for species inhabiting arid regions where moisture is often a limiting factor (Yang *et al*., 2012). We have shown that the dispersal distance of M^+^ seeds is significantly restricted in wet conditions (Figs 5, 6). M^+^ seeds have the capacity to travel only short distances via runoff water (Fig. 6a). Our findings, therefore, support the view that myxospermy provides an antitelechoric mechanism by retaining the M^+^ seed in favourable microclimates. Interactions with substrata provide a further means of adhesion and retention (Fig. 5). The relative increase in mass of the dry M^+^ seed, in particular when exposed to wet sand, illustrates the effectiveness of antitelechoric seed coat mucilage production (Grubert, 1974). This repression of dispersal is an adaptive mechanism, but it does not exclude rare cases of long‐distance transport by exozoochory (Mummenhoff & Franzke, 2007). Dispersal of the M^+^ seed diaspore therefore allows persistence in relatively stable environments by repeated establishment in few favourable sites. Nondispersed seeds may remain enclosed within DEH fruits and their spatiotemporal dispersal may be staggered by later rain events. Further analyses combining ecophysiological and genetic tools will shed light on the role and evolutionary advantages of seed coat mucilage production in *A. arabicum*.

### Adaptive features of IND diaspores support telechory

In contrast to the M^+^ seed, the adaptive features of the deeper dormant IND fruit morph promote telechory by wind and water. The convergent evolution of indehiscence within the Brassicaceae was associated with the evolution of pericarp features that enhance dispersal as well as an abscission zone on the joint between fruit segments in *Cakile* (Willis *et al*., 2014). Fruit traits associated with greater dispersal ability, that is, indehiscence plus pericarp features, were also associated with the evolution of larger seeds. In agreement with a greater dispersal ability (compared with the M^+^ seed), the *A. arabicum* IND fruit morph is indeed characterized by pericarp features that enhance dispersal, but it is not associated with an increased M^−^ seed size (Table 1). The IND fruit morph also does not contain a joint, but the abscission zone that attaches it to the plant is well developed (Lenser *et al*., 2016). In agreement with its dispersal by abscission (Fig. 1a), the force required to remove IND fruits from *A. arabicum* plants is approximately sixfold lower than that required for DEH fruits in dry conditions, and *c*. 16‐fold lower than that for abscission triggered by rain (ombrohydrochory). This allows wind dispersal by abscission of IND fruits in dry conditions late in summer, and a further enhanced IND fruit abscission in wetter conditions in autumn. Consistent with supporting telechoric mechanisms is a wider wind dispersal kernel and a > 500‐fold greater buoyancy of IND fruits compared with M^+^ seeds.

Although plant species with anemochorous diaspores exhibit a cosmopolitan distribution, wind dispersal in itself is regarded as a derived dispersal mechanism (van der Pijl, 1982). Structurally, the wings of the IND pericarp confer rigidity with a large surface area and low mass (Table 1). Wing‐loading for IND fruits is therefore relatively low (data not shown). The fruit valves of the pericarp comprise anatomically dead tissue filled with air which is fully permeable for water. However, the chemical constitution of the M^−^ seed coat, presence of small air pockets in the outer walls of the epidermal cells, or hydrophobic properties of the pericarp may prevent excessive moisture absorbance and confer enhanced buoyancy properties (Fig. 5b). Observations of mature infructescences during humid conditions suggest that IND fruit abscission may not be the first step in its dispersal; moisture‐induced movements (hygrochasy) of fruit pedicels (Lenser *et al*., 2016) facilitate maximal exposure to forces enabling abscission and dispersal by wind and rain. This mechanism, present in a number of desert annuals, may correlate diaspore dispersal to rain events, which ensure optimal germination conditions (Gutterman, 1993). In addition, the presence of densely cytoplasmic cells at the base of the fruit–pedicel junction in the IND fruit morph allows programmed abscission in response to such developmental and environmental cues. All these properties are consistent with IND fruit adaptations for telechoric dispersal and may be interpreted as a more opportunistic strategy to permit longer‐distance range dispersal, including over the hilly terrain in the native habitat of *A. arabicum*.

### Ecological significance of *A. arabicum* diaspore dimorphism

A complex evolutionary interdependence between dormancy and dispersal influences population structure and demography via interactions among multiple traits and selective processes (de Casas *et al*., 2015). The dimorphic seed dispersal strategy in *A. arabicum* represents a fascinating tradeoff between promoting telechory (IND fruit diaspores) and antitelechory (M^+^ seed diaspores). As in most described heteromorphic systems, in *A. arabicum* one of the diaspores (IND fruit) has a high degree of dormancy (HDo, i.e. low germinability), whereas the other diaspore (M^+^ seed) has a low degree of dormancy (LDo). The observed difference in germinability is consistent with our finding that the IND fruit (HDo) has a higher ABA concentration than does the M^+^ seed (LDo). Interestingly, whereas in most systems the HDo is combined with a low dispersal ability (LDi) and the LDo with a high dispersal ability (HDi) (Lu *et al*., 2013, 2015; Baskin *et al*., 2014), our biophysical and biochemical analysis of abiotic dispersal and dormancy properties revealed that this is different in *A. arabicum*. We found that the more abundant, myxospermous M^+^ seed diaspore combines LDi–LDo, while the IND fruit diaspore combines HDi–HDo. The altered dispersal and dormancy properties of the IND fruit morph are almost exclusively conferred by the distinct pericarp features and the high ABA concentration of the IND diaspore.

Dispersal and dormancy provide two bet‐hedging strategies which can evolve under fluctuations in the environmental conditions in space and time (Volis & Bohrer, 2013; de Casas *et al*., 2015). There is extensive theoretical literature on this subject, from which the general picture emerges in many cases that these strategies are negatively associated (Buoro & Carlson, 2014; de Casas *et al*., 2015). One of these two strategies tends to be dominant: high dispersal associates with low dormancy, and low dispersal with high dormancy. However, much of this depends on the details of the models used. A further selective force emerges from the effects of local competition and the inclusive fitness effects that this brings. For example, in a structured deme model, in which dormancy and dispersal are allowed to evolve together, selection favours nondispersing seeds to have low dormancy (Vitalis *et al*., 2013). A possible explanation for the evolution of HDo/HDi morphs was hypothesized by Buoro & Carlson (2014) and de Casas *et al*. (2015), who argued that the joint evolution of dispersal and dormancy can be explained by environmental correlation. Spatially uncorrelated environments lead to high dispersal, and temporally uncorrelated environments to high dormancy. If indeed environments in the natural habitat of *A. arabicum* are both spatially and temporally uncorrelated, this might explain the observed pattern for the IND morph: high dormancy coupled with high dispersal.

A further, more empirically grounded, explanation is that, in *A. arabicum*, the seed ontology links HDi to HDo. The altered dispersal and dormancy properties of the IND fruit morph are almost exclusively conferred by the distinct pericarp features. In many fruit diaspores, the evolution of HDi pericarp features is associated with an increased seed size (Willis *et al*., 2014), but we found that this is not the case in *A. arabicum*. If selection for high dispersal variants is the dominant force, it will then go together with high dormancy. The LDi–LDo M^+^ seed and HDi–HDo IND fruit diaspores may therefore have evolved as an adaptation to semiarid habitats with varied topography, which creates microclimates by elevation in mountain belts such as the South Anatolian Taurus (Apaydin *et al*., 2011).

Mechanistic modelling of diaspore dispersal by wind over hilly terrain revealed that even gentle topography introduced considerable variability in the distance and direction of dispersal as a result of local turbulences (Trakhtenbrot *et al*., 2014). Most alpine plant species have a limited capacity for diaspore dispersal beyond 10 m, and time their germination and seedling emergence with seasonal temperature regimes (Ohsawa *et al*., 2007; Mondoni *et al*., 2012). The winged, symmetrical fruit valve membranes, together with a localized concentration of mass (M^−^ seed), contribute to high IND fruit dispersal ability in air currents. Thus, through the act of thermal convection currents and air flows typically experienced in scree slope habitats, a vertical up‐current may result in a large fall time for such a winged diaspore. This contrasts with the mother‐site (or safe‐site) theory, originally proposed by Zohary (1937), which predicts the putative low benefit of dispersal in harsh and unpredictable environments, where dispersal and repeated establishment in local favourable sites ensure persistence of plant species and populations, but rather suggests a species that is persisting in linked sink habitats through dispersal between these sinks (Jansen & Yoshimura, 1998).

Our working hypothesis is that the plasticity of *A. arabicum* to alter the ratios and numbers of the dimorphic diaspores in response to temperature during the reproductive phase, combined with the anemochorous dispersal ability of the IND fruit diaspore, supports the longer‐distance dispersal over hilly terrain. In mountainous environments, there is considerable variation in habitat at a relatively small scale: plants at higher altitude tend to be in a more exposed, harsh and unpredictable environment that is comparatively devoid of intense competition. Ambient temperature may act as a reliable clue to a habitat's altitude, thus allowing plants to sense which habitat they are in; by altering the ratio of seed morphs with temperature, *A. arabicum* can adjust its dispersal strategy to risks and fluctuations in the differing habitat conditions. Future ecological work in the field is required to test this hypothesis by analysing the phenology of *A. arabicum* seedling emergence and the relative numbers of the distinct dimorphic diaspores in relation to elevation.

### Conclusions

The fascinating morphological, biophysical, hormonal and ecological adaptations of the diaspore dimorphism in *A. arabicum* reveal that they support telechory and antitelechory as contrasting dispersal strategies. The *A. arabicum* dimorphic diaspore system is distinct from most other heteromorphic species in several key features. First, it exhibits plasticity in response to the reproduction temperature in producing distinct numbers and ratios of the dimorphic diaspores. Second, for the myxospermous M^+^ seed diaspore, low dispersal ability is combined with low dormancy (LDi–LDo) to support antitelechory. Third, for the IND fruit diaspore, high dispersal ability is combined with high dormancy (HDi–HDo) to support telechory by wind and water as dispersal agents. Furthermore, these key differences in the dispersal ability and germinability of the dimorphic diaspores are conferred on the IND fruit diaspore by specific pericarp‐derived features and high ABA concentration. Only the IND fruit diaspore can provide longer‐range dispersal by wind and water. We propose that the unique features of the *A. arabicum* diaspore dimorphism and its phenotypic plasticity evolved as a bet‐hedging adaptation for survival in semiarid habitats and high elevational scree‐slope environments.

## Author contributions

KM, KS, WA, TS and GL‐M planned and designed the research; KS, WA and TS performed experiments; WA, KS, TS, GL‐M and KM analysed and interpreted the data; BN and VAAJ formulated the hypothesis that temperature provides the cue for elevation, leading to adaptive plasticity of diaspore ratio; WA, TS, GL‐M and KM wrote the manuscript; all authors revised and approved the final article. WA, KS, GL‐M and KM contributed equally to this work.
